# A Simple Quality Assessment Index for Stereoscopic Images Based on 3D Gradient Magnitude

**DOI:** 10.1155/2014/890562

**Published:** 2014-07-15

**Authors:** Shanshan Wang, Feng Shao, Fucui Li, Mei Yu, Gangyi Jiang

**Affiliations:** Faculty of Information Science and Engineering, Ningbo University, Ningbo 315211, China

## Abstract

We present a simple quality assessment index for stereoscopic images based on 3D gradient magnitude. To be more specific, we construct 3D volume from the stereoscopic images across different disparity spaces and calculate pointwise 3D gradient magnitude similarity (3D-GMS) along three horizontal, vertical, and viewpoint directions. Then, the quality score is obtained by averaging the 3D-GMS scores of all points in the 3D volume. Experimental results on four publicly available 3D image quality assessment databases demonstrate that, in comparison with the most related existing methods, the devised algorithm achieves high consistency alignment with subjective assessment.

## 1. Introduction

In recent years, there has been great progress in developing objective image quality assessment (IQA) metrics [1]. However, the development of 3D image/video quality index is still in its early stage. Assessing the 3D image quality is a very challenging issue because it is affected by 2D image quality, depth perception, visual comfort, and other factors [2, 3]. It is particularly challenging when the stereoscopic image pair consists of two views with different quality levels. Therefore, how to understand the binocular vision perception, for example, binocular rivalry in stereosis [4], is still limited in 3D image quality assessment (3D-IQA).

Numerous approaches for full-reference 2D image quality assessment (2D-IQA) have been widely researched over the last several decades, such as structural similarity (SSIM) [5], multiscale SSIM (MS-SSIM) [6], and UQI (universal quality index) [7]. Among these 2D metrics, gradient information has been employed in various ways. Chen et al. [8] proposed a gradient SSIM (G-SSIM) metric based on the edge as the structure information. Liu et al. [9] devised an IQA approach by integrating gradient similarity and luminance similarity. Zhu and Wang [10] proposed a multiscale visual gradient similarity (VGS) model by adopting different properties of gradient. Xue et al. [11] proposed a new effective gradient magnitude similarity deviation (GMSD) model to predict the overall image quality score. However, 3D-IQA is still a less investigated problem due to lack of understanding of 3D visual perception. In this paper, we simply classify the existing 3D-IQA into the following two categories: (1) evaluate stereoscopic images using 2D-IQA metrics; (2) evaluate stereoscopic images considering 3D perceptual properties.

The most direct way of applying state-of-the-art 2D-IQA methods to 3D-IQA is to evaluate the two views of the stereoscopic images, disparity/depth image, separately by 2D metrics, and then combine them into an overall score. Boev et al. [12] combined monoscopic and stereoscopic quality components from the “Cyclopean” image and disparity map, respectively, for stereo-video evaluation. Campisi et al. [13] computed quality scores of both stereo-pair and the disparity map by 2D quality metrics and then combined them to produce a final score. You et al. [14] investigated various 2D quality evaluators on a stereo-pair and its disparity map and found the optimal combination which can yield the best performance. Hewage et al. [15] investigated the effectiveness of three 2D metrics (PSNR, VQM, and SSIM) to predict the perceived quality of compressed color plus depth 3D video. However, for effective 3D evaluation, we cannot assess the perceived quality directly using 2D-IQA metrics (factors toward the perceived quality are different in 3D).

For measuring the perceived quality of stereoscopic images, several metrics have been proposed by integrating 3D perceptual properties. Hwang and Wu [16] fused the impacts of visual attention, depth variation, and stereo distortion in the stereo image quality assessment. Bensalma and Larabi [17] devised a binocular energy quality metric (BEQM) by modeling the complex cells responsible for the construction of the binocular energy. Chen et al. [18] constructed a “Cyclopean” image from the stereo-pair and evaluated the quality of “Cyclopean” image by 2D-IQA metrics. De Silva et al. [19] measured the quality of symmetrically and asymmetrically compressed artifacts by quantifying structural distortion, asymmetric blur, and content complexity. In our previous work [20], we proposed a perceptual quality assessment metric by considering binocular visual characteristics, in which the stereoscopic images are separated into noncorresponding, binocular fusion, and binocular suppression regions. Other relevant works can be found in [21–24].

In this paper, we proposed a simple yet effective quality assessment index for stereoscopic images based on 3D gradient magnitude. The main contributions of this paper are as follows: (1) we construct 3D data from a stereoscopic image pair to account for depth perception under different disparity spaces; (2) we compute 3D gradient using different kernels on horizontal, vertical, and viewpoint directions; (3) we demonstrate that 3D gradient magnitude allows more emphasis on distortions around edge regions in the proposed 3D-IQA scheme. The rest of the paper is organized as follows.  Section 2 presents 3D data construction.  Section 3 presents the proposed IQA for stereoscopic images. The experimental results are given and discussed in Section 4, and, finally, conclusions are drawn in Section 5.

## 2. 3D Data Construction

As known, the process of binocular visual perception is regarded as responses of a pair of simple cells received from the left and right eyes [25]. The output of a simple receptive field at a position (*x*, *y*) is formulated as convolution with a filter function *g*() (e.g., Gabor filter):

(1) $$\begin{array}{c} C_{v}\left(x,y\right)=\overset{+\infty }{\underset{-\infty }{\iint }}g_{v}\left(x-\xi ,y-\eta \right)I\left(\xi ,\eta \right)d\xi \;d\eta . \end{array}$$

Then, binocular energy response combines the output of the receptive fields of both left and right images as [26]

(2) Ev=||Clv+Crv||2=(Re[Clv]+Re[Crv])2+(Im⁡[Clv]+Im⁡[Crv])2,

where *Re*[] and *Im*⁡[] are real and imaginary parts of the response. With this understanding, the preferred disparity can be estimated by *D* = Δ*ϕ*_*lr*_/*ω*, where Δ*ϕ*_*lr*_ = *ϕ*_*l*_ − *ϕ*_*r*_ is the phase difference between the left and right images, *ϕ*_*r*_ = arctan(*Im*⁡(*C*_*rv*_)/*Re*(*C*_*rv*_)), *ϕ*_*l*_ = arctan(*Im*⁡(*C*_*lv*_)/*Re*(*C*_*lv*_)), and *ω* is the radial frequency of the cell.

Depth perception is the most important feature for stereoscopic images, which occurs as a result of the horizontal separation between the left and right eyes [27]. The different locations on the two cells are crucial to detect variations in depth. Given two input images, *I*_*L*_(*x*, *y*) and *I*_*R*_(*x*, *y*), the goal of disparity estimation is to find an optimal binocular disparity *d*_*L*_(*x*, *y*) so that the two images match as closely as possible:

(3) $$\begin{array}{c} I_{L}\left(x,y\right)≅I_{R}\left(x-d_{L}\left(x,y\right),y\right). \end{array}$$

An important issue for understanding the binocular vision is how to characterize binocular disparity. However, it is usually not easy to assess the quality of the estimated disparity since ground truth disparity is generally not available. Numerous disparity estimation algorithms had been proposed [28, 29]. Therefore, we define disparity space image (DSI) as the squared difference between the shifted left and right images as follows [30]:

(4) $$\begin{array}{c} \text{DSI}\left(x,y,d\right)={\left(I_{L}(x,y)-I_{R}(x-d,y)\right)}^{2}. \end{array}$$

**Figure 1:**
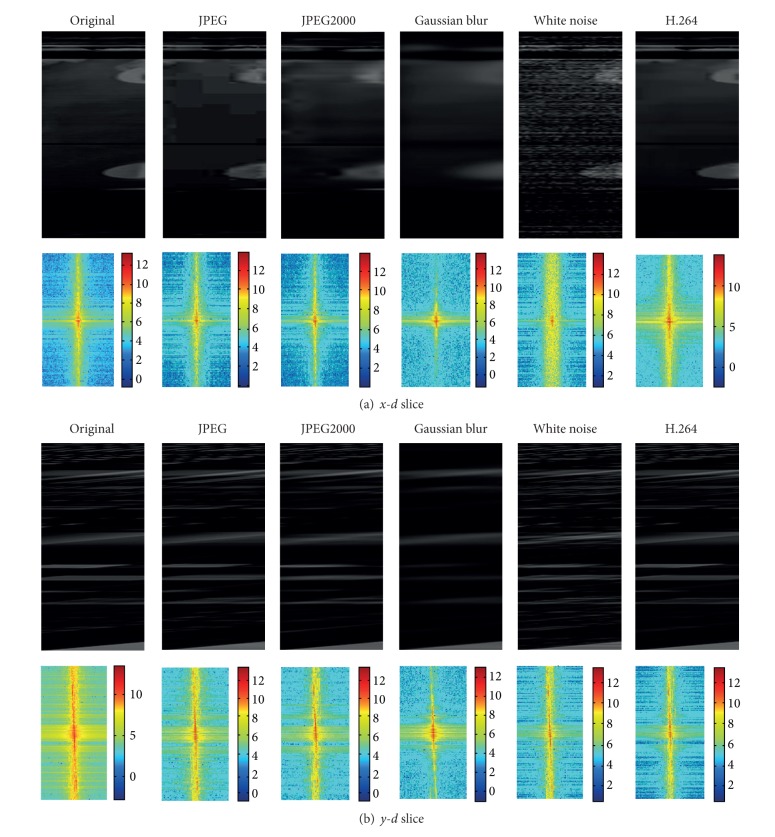
The figure of* x*-*d* and* y*-*d* cross-sectional views under different types of distortion.

Thus, we can obtain a 3D volume of intensity differences over the spatial positions and the disparity ranges. The disparity can be obtained by searching the optimal path from the 3D volume. In this paper, we advocate the 3D volume as the basic processing unit. The local structured features in the DSI can effectively reflect the impact of distortion on different disparity ranges. Therefore, it is useful to think about the quality assessment issue by adding some types of distortion across different disparity spaces.  Figure 1 shows the different slice sampling of the DSI under different types of distortion. It is obvious that quality degradation in the left and right views will be directly reflected by the computed DSI; that is, the disparity values with the minimum DSI values are not the same before and after degradation; thus, depth perception will be affected (i.e., it can be measured by the DSI).

## 3. Proposed Quality Assessment Index

### 3.1. Traditional SSIM Index

The SSIM index in [5] is defined as the similarity of three components: luminance similarity, contrast similarity, and structural similarity, and these three components are mathematically described as

(5) l(x,y)=2μxμy+C1μx2+μy2+C1c(x,y)=2σxσy+C2σx2+σy2+C2s(x,y)=2σxy+C3σxσy+C3,

where *μ*_*x*_, *μ*_*y*_, *σ*_*x*_^2^, *σ*_*y*_^2^, and *σ*_*xy*_ are the mean of *x*, the mean of *y*, the variance of *x*, the variance of *y*, and the covariance of *x* and *y*, respectively; *C*_1_, *C*_2_, and *C*_3_ are constants to avoid the denominator being zero. The above results range in [0,1], in which 0 indicates no similarity between two numbers and 1 implies perfect similarity between two numbers. The SSIM index is given as

(6) $$\begin{array}{c} \text{SSIM}\left(x,y\right)={\left[l\left(x,y\right)\right]}^{\alpha }{\left[c\left(x,y\right)\right]}^{\beta }{\left[s\left(x,y\right)\right]}^{\gamma }, \end{array}$$

where *α*, *β*, and *γ* are parameters to adjust the relative importance of three components. In this work, we generalize the single-image SSIM index to a new 3D image pair quality index by incorporating 3D gradient magnitude information.

### 3.2. 3D Gradient Computation

**Figure 2:**
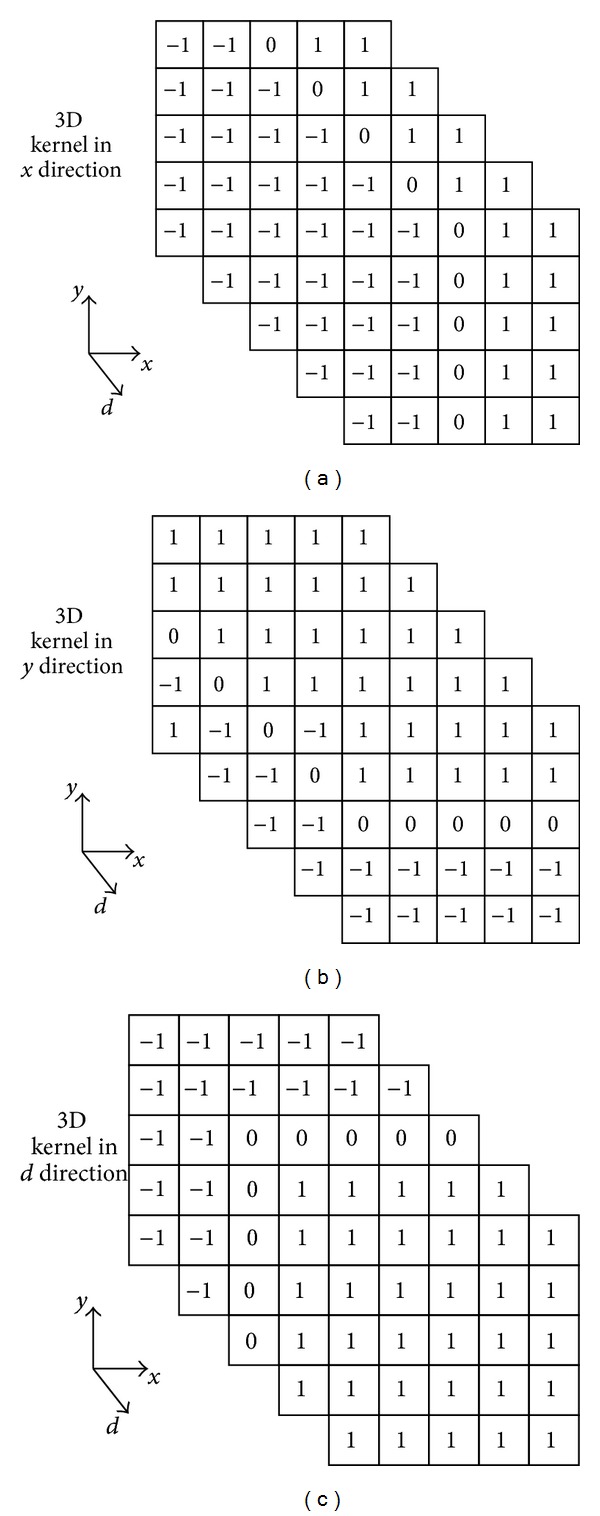
Kernels used for 3D gradient computation in three directions.

In 2D image, the gradient is usually computed by convolving an image with a linear filter, such as Roberts, Sobel. In this work, we use different kernels to compute the 3D gradient on three directions. For simplicity, we use the kernels in [31] with first order of derivative shown in Figure 2. Since the nonzero elements' absolute values are 1, convolving the kernels with a 3D volume yields the horizontal, vertical, and viewpoint gradients that can be fast computed by

(7) $$\begin{array}{c} \nabla f\left(x,y,d\right)=\left[\begin{array}{c} \overset{j=d+2}{\underset{j=d-2}{\sum }}\text{Con}X\left(x,y,j\right)\\ \overset{j=d+2}{\underset{j=d-2}{\sum }}\text{Con}Y\left(x,y,j\right)\\ \overset{j=d+2}{\underset{j=d-2}{\sum }}\mathrm{sign}\left(j-d\right)\text{Con}Z\left(x,y,j\right) \end{array}\right], \end{array}$$

where

(8) ConX(x,y,j)=−∑v=y−2u=x−2v=y+2u=x−1f(u,v,j)+∑v=y−2u=x+1v=y+2u=x+2f(u,v,j)ConY(x,y,j)=−∑v=y−2u=x−2v=y−1u=x+2f(u,v,j)+∑v=y+1u=x−2v=y+2u=x+2f(u,v,j)ConZ(x,y,j)=−∑v=y−2u=x−2v=y+2u=x+2f(u,v,j).

### 3.3. 3D Gradient Magnitude Similarity (3D-GMS) Based Quality Metric

With the 3D gradient magnitude values of the original and distorted 3D volumes, the 3D-GMS index is defined as

(9) $$\begin{array}{c} 3\text{D-GMS}=\frac{1}{N}\underset{d}{\sum }\;\underset{\left(x,y\right)}{\sum }\frac{2m_{o}\left(x,y,d\right)\cdot m_{d}\left(x,y,d\right)+C_{4}}{m_{o}^{2}\left(x,y,d\right)+m_{d}^{2}\left(x,y,d\right)+C_{4}}, \end{array}$$

where the parameter *C*_4_ is a constant to avoid the denominator being zero; *m*_*o*_(*x*, *y*, *d*) and *m*_*d*_(*x*, *y*, *d*) are the 3D gradient magnitudes of the original and distorted 3D volumes, which are defined as the root mean square of directional gradients along three directions:

(10) $$\begin{array}{c} m_{o}\left(x,y,d\right)=\sqrt{{\left(\nabla f_{x}^{o}\right)}^{2}+{\left(\nabla f_{y}^{o}\right)}^{2}+{\left(\nabla f_{d}^{o}\right)}^{2}}\\ m_{d}\left(x,y,d\right)=\sqrt{{\left(\nabla f_{x}^{d}\right)}^{2}+{\left(\nabla f_{y}^{d}\right)}^{2}+{\left(\nabla f_{d}^{d}\right)}^{2}}. \end{array}$$

**Figure 3:**
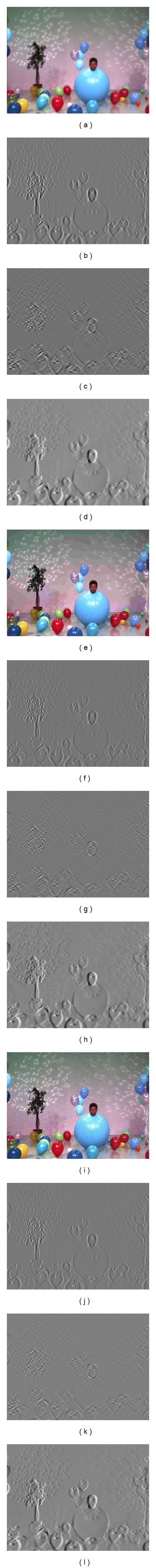
Examples of quality degraded left images and the corresponding gradient maps of “Balloons” test sequence. (a)~(d): (a) Gaussian blurred image; (b) horizontal gradient map of (a); (c) vertical gradient map of (a); (d) viewpoint gradient map of (a). DMOS = 29.435, 3D-GMS = 0.9720; (d)~(g): (e) JPEG compressed image; (f) horizontal gradient map of (e); (g) vertical gradient map of (e); (h) viewpoint gradient map of (e). DMOS = 30.609, 3D-GMS = 0.9803; (i)~(l): (i) WN distorted image; (j) horizontal gradient map of (i); (k) vertical gradient map of (i); (l) viewpoint gradient map of (i). DMOS = 30.130, 3D-GMS = 0.9793.

The 3D-GMS value reflects the range of distortion degrees in an image. The higher the 3D-GMS value, the larger the distortion rang, and, thus, the lower the image perceptual quality. Here, we present one example to illustrate this point above. The first row of Figure 3 shows (a) Gaussian blurred image of “Balloons” test sequences from NBU IQA database and the corresponding horizontal, vertical, and viewpoint gradient maps in (b)~(d). The second row of Figure 3 shows the JPEG compressed image in (e) and the corresponding horizontal, vertical, and view gradient maps in (f)~(h). The third row of Figure 3 shows the white noise (WN) distorted image in (i) and the corresponding horizontal, vertical, and view gradient maps in (j)~(l). Note that only one selected viewpoint is selected for the viewpoint gradient maps in (d), (h), and (l). The difference mean opinion scores (DMOS) values for the Gaussian blurred, JPEG compressed, and WN distorted stereoscopic images are 29.435, 30.609, and 30.130, respectively; that is, the subjective measures for these distorted stereoscopic images are similar. The 3D-GMS scores for these distorted stereoscopic images are 0.9720, 0.9803, and 0.9793, respectively. It is clearly demonstrated that the quality scores are more consistent with the DMOS values.

## 4. Experimental Results and Analyses

### 4.1. Databases and Performance Measures

In the experiment, four publicly available 3D IQA databases: NBU 3D IQA Database [20], LIVE 3D IQA Phase I Database [18], and LIVE 3D IQA Phase II Database (including symmetric and asymmetric databases) [32] are used to verify the performance of the proposed metric for stereoscopic images. The NBU 3D IQA Database consists of 312 distorted stereoscopic pairs generated from 12 reference stereoscopic images. Five types of distortions, JPEG, JP2K, Gblur, WN, and H.264, are symmetrically applied to the left and right reference stereoscopic images at various levels. The LIVE 3D IQA Phase I Database consists of 365 distorted stereoscopic pairs generated from 20 reference stereoscopic images. The LIVE 3D IQA Phase II-Symmetric Database and Phase II-Asymmetric Database consist of 210 and 240 distorted stereoscopic pairs generated from 8 reference stereoscopic images, respectively. Five types of distortions, JPEG, JP2K, Gblur, WN, and FF, are symmetrically applied to the left and right reference stereoscopic images at various levels for the LIVE 3D IQA Phase I Database and LIVE 3D IQA Phase II-Symmetric Database and asymmetrically applied for the LIVE 3D IQA Phase II-Asymmetric Database.

In the paper, three commonly used performance indicators are used to benchmark the proposed metric against the relevant state-of-the-art techniques: Pearson linear correlation coefficient (PLCC), Spearman rank order correlation coefficient (SRCC), and root mean squared error (RMSE), between the objective and subjective scores. For a perfect match between the objective and subjective scores, PLCC = SRCC = 1 and RMSE = 0. For the nonlinear regression, we use the following five-parameter logistic function [33]:

(11) $$\begin{array}{c} {\text{DMOS}}_{p}=\beta_{1}\cdot \left(\frac{1}{2}-\frac{1}{1+\exp\left(\beta_{2}\cdot \left(x-\beta_{3}\right)\right)}\right)+\beta_{4}\cdot x+\beta_{5}, \end{array}$$

where *β*_1_, *β*_2_, *β*_3_, *β*_4_, and *β*_5_ are determined by using the subjective scores and the objective scores.

### 4.2. Overall Assessment Performance

**Figure 4:**
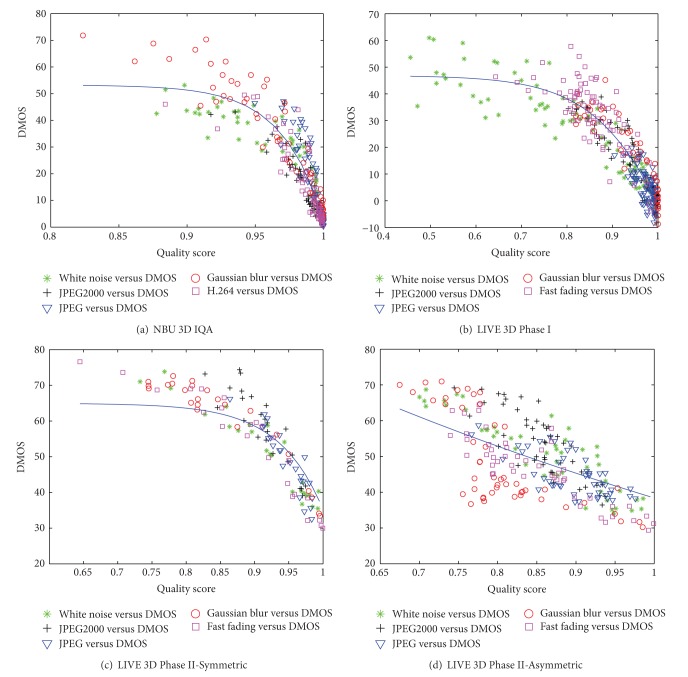
Scatter plots of predicted quality scores against the subjective scores (DMOS) of the proposed method on four databases.

In Table 1, we compare the competing 2D-IQA and 3D-IQA metrics' performance on the four databases in terms of PLCC, SRCC, and RMSE. For the three 2D-IQA metrics, they directly estimate the quality of each view separately and generate a weighted average score. The proposed scheme outperforms the three 2D-IQA schemes in the databases. For You et al.'s and Benoit et al.'s schemes, since they are the combination of 2D image quality metrics for stereoscopic images and disparity maps, the performance of the two schemes is highly dependent on the estimated disparity maps (stereo matching algorithm [29] is used in this paper), and the proposed scheme performs better than the two schemes on three databases (i.e., NBU 3D IQA Database, LIVE 3D IQA Phase I Database, and LIVE 3D IQA Phase II-Symmetric Database with symmetrical distortions). The performances of Bensalma et al.'s, Chen et al.'s, and Shao et al.'s schemes are reasonably good on most of the databases, but the proposed scheme can still get comparable performance.  Figure 4 shows the scatter plots of predicted quality scores against subjective quality scores (in terms of DMOS) of the proposed scheme on the three databases. Overall, the proposed scheme has an impressive consistency with human perception.

### 4.3. Performance Comparison on Individual Distortion Types

To more comprehensively evaluate the prediction performance of the proposed method, we compare the nine schemes on each type of distortion. The PLCC and SRCC results are listed in Tables 2 and 3, where the top two metrics have been highlighted in boldface. One can see that the proposed scheme is among the top 2 metrics 13 times in terms of PLCC, followed by You et al.'s scheme (among the top 2 metrics 9 times), Shao et al.'s scheme (among the top 2 metrics 6 times). However, the overall performance of You et al.'s and Shao et al.'s scheme is not the best on the four databases. Since the proposed scheme is to measure the structure degradation, it is especially for Gblur distortion type and is an effective measure for WN distortion type on the NBU 3D IQA Database, LIVE 3D IQA Phase I Database, and LIVE 3D IQA Phase II-Symmetric Database. Even though some 2D metrics may have remarkable performances in evaluating the qualities of 2D images, they may not be sufficient to predict the perceptual quality of stereoscopic images. In general, the proposed 3D gradient magnitude can serve as an excellent feature for quality prediction.

### 4.4. Discussion of Computational Complexity

Computational complexity is another important factor to evaluate the performance of the proposed scheme. The DSIs are computed offline in advance. The main operations in the proposed 3D-GMS include calculating 3D gradients (by convolving three different 5 × 5 × 5 templates), thereby producing gradient magnitude maps. Overall, the proposed 3D-GMS can provide a low-complexity solution for 3D-IQA, compared with these 3D-IQA metrics (e.g., You et al.'s, Benoit et al.'s, Bensalma et al.'s, Chen et al.'s, and Shao et al.'s schemes).

## 5. Conclusions

In this study, we devised a simple yet effective quality assessment index, called 3D gradient magnitude similarity (3D-GMS), for stereoscopic images. More specifically, we construct 3D volume from the stereoscopic images across different disparity spaces and calculate pointwise gradient magnitude similarity along three directions. Then, average 3D-GMS score for all points in the 3D volume is computed as the final quality index. Compared with state-of-the-art 2D image quality assessment (2D-IQA) and 3D image quality assessment (3D-IQA) metrics, the proposed 3D-GMS metric performs better in terms of both accuracy and efficiency on four publicly available 3D IQA databases. In the future work, we will further explore how to combine 3D visual perceptual models, such as 3D visual attention, into the 3D-GMS metric.

## Figures and Tables

**Table 1:** Performance of the proposed method and the other seven schemes in terms of PLCC, SRCC, and RMSE on the four databases (the cases in bold: the best performance).

IQA model	NBU (312 images)			LIVE I (365 images)			LIVE II-S (120 images)			LIVE II-A (240 images)		
	PLCC	SRCC	RMSE	PLCC	SRCC	RMSE	PLCC	SRCC	RMSE	PLCC	SRCC	RMSE
PSNR	0.8255	0.8519	9.6960	0.8354	0.8339	9.0117	0.7651	0.7768	8.0389	0.6659	0.6752	7.5610
SSIM	0.8347	0.8575	9.4582	0.8887	0.8873	7.5155	0.7765	0.7488	7.8656	**0.7676**	**0.7388**	**6.4949**
MS-SSIM	0.8510	0.9295	9.0213	**0.9287**	**0.9224**	**6.0771**	0.8824	0.9077	**5.8782**	0.7329	0.7093	6.8947
Benoit [13]	0.7838	0.8118	10.6675	0.8786	0.8852	7.8281	0.8312	0.8412	6.9411	0.7622	**0.7342**	6.5613
You [14]	0.8205	0.8246	9.8196	0.9172	0.9248	6.5328	**0.9190**	**0.9491**	**4.9206**	0.7469	0.7184	6.7388
Bensalma [17]	0.9378	0.9381	5.9615	0.8902	0.8746	7.4683	0.8539	0.8418	6.4956	**0.7663**	0.7210	**6.5111**
Chen [18]	**0.9388**	**0.9374**	**5.9153**	0.9220	0.9078	6.3474	0.8511	0.8624	6.6044	0.6317	0.6301	7.9343
Shao [20]	0.9266	0.9271	6.4597	0.9270	0.9217	6.1497	0.9286	0.9153	4.6323	0.6098	0.6300	8.0329
Proposed	0.9240	0.9331	6.5711	0.9213	0.9158	6.3748	**0.9515**	**0.9443**	**3.8411**	0.7277	0.6951	6.9520

**Table 2:** Performance comparison of the eight schemes on each individual distortion type in terms of PLCC.

	Criteria	PSNR	SSIM	MS-SSIM	Benoit [13]	You [14]	Bensalma [17]	Chen [18]	Shao [20]	Proposed
NBU	JPEG	0.7851	0.8538	0.9362	0.8062	0.7996	0.8926	**0.9334**	**0.9378 **	0.9310
	JP2K	0.6960	0.8201	0.9103	0.7312	0.7775	**0.9442**	**0.9513**	0.9192	0.9223
	Gblur	0.8690	0.9254	0.8990	0.8760	0.9364	0.9599	0.8938	**0.9608**	**0.9616**
	WN	**0.9549**	0.9362	0.8659	0.9316	0.8749	0.8961	0.9466	0.9447	**0.9562**
	H246	0.7965	0.8808	0.9359	0.7506	0.8197	**0.9525**	**0.9604**	0.9269	0.9274

LIVE I	JPEG	0.1982	0.4955	0.5906	0.4773	0.6216	0.3762	0.4756	**0.6845 **	**0.6657**
	JP2K	0.7889	0.8683	0.8690	0.8762	**0.9376**	0.8484	0.8553	0.9081	**0.9326**
	Gblur	0.8497	0.9119	0.9432	0.9180	**0.9538**	0.9157	0.9384	**0.9525 **	0.9400
	WN	0.9394	0.9378	0.9147	0.9159	0.9350	0.9136	**0.9532**	**0.9521**	0.9259
	FF	0.6997	0.6926	0.8001	0.7393	**0.8496**	0.7233	0.7969	**0.8421 **	0.8069

LIVE II-S	JPEG	0.2967	0.6769	0.8127	0.8308	**0.8720**	0.3474	0.6012	0.8450	**0.9314**
	JP2K	0.5839	0.8161	0.8334	0.8323	**0.9203**	0.6896	0.6703	0.8954	**0.9211**
	Gblur	0.8706	0.8324	0.9322	0.9256	**0.9779**	0.9526	0.9178	0.8991	**0.9845**
	WN	0.9187	**0.9749**	0.9688	0.9591	0.9371	0.9359	0.9462	0.9654	**0.9667**
	FF	0.8135	0.8622	0.9128	0.9321	**0.9806**	0.9164	0.9382	0.9641	**0.9774**

LIVE II-A	JPEG	0.5488	0.6847	0.8078	0.7162	0.7036	0.6273	0.5347	0.6523	**0.7952**
	JP2K	0.6448	0.7359	0.7925	0.7659	**0.8684**	0.6771	0.6540	0.7824	**0.8395**
	Gblur	0.8442	0.7391	0.7556	0.8195	**0.9719**	0.8621	0.6918	0.7725	**0.8742**
	WN	0.8077	0.9112	**0.9404**	0.8635	0.8935	0.9236	**0.9379**	0.7820	0.6919
	FF	0.7522	0.8662	0.8485	0.8656	0.7584	**0.8805**	0.8138	0.7819	**0.8992**

**Table 3:** Performance comparison of the eight schemes on each individual distortion type in terms of SRCC.

	Criteria	PSNR	SSIM	MS-SSIM	Benoit [13]	You [14]	Bensalma [17]	Chen [18]	Shao [20]	Proposed
NBU	JPEG	0.8808	0.8770	**0.9505**	0.8218	0.8275	0.9148	**0.9555**	0.9489	0.9379
	JP2K	0.8827	0.8528	0.9420	0.7710	0.7676	**0.9508**	**0.9456**	0.9309	0.9434
	Gblur	0.9331	0.9324	**0.9695**	0.8847	0.9347	0.9559	**0.9691**	0.9510	0.9609
	WN	0.9278	0.8816	0.9009	0.8882	0.8363	0.9157	0.9096	**0.9336**	**0.9436**
	H246	0.8716	0.8671	**0.9493**	0.7652	0.7880	0.9379	**0.9502**	0.9470	0.9349

LIVE I	JPEG	0.2048	0.4554	0.5992	0.4755	0.6034	0.3282	0.4349	**0.6148 **	**0.6342**
	JP2K	0.8010	0.8669	0.8890	0.8667	**0.8983**	0.8170	0.8712	0.8752	**0.8938**
	Gblur	0.9019	0.8985	**0.9241**	0.8790	**0.9322**	0.9179	0.9208	0.9375	0.9120
	WN	0.9316	0.9378	**0.9435**	0.9388	0.9396	0.9054	0.9386	**0.9431 **	0.9233
	FF	0.5874	0.6254	0.7293	0.6105	**0.8172**	0.6500	0.7477	**0.7814 **	0.7391

LIVE II-S	JPEG	0.3231	0.7179	0.8432	0.8156	**0.8939**	0.4996	0.6304	0.8287	**0.9285**
	JP2K	0.5547	0.7260	0.7826	0.8043	0.8956	0.6078	0.6617	**0.9148 **	**0.9026**
	Gblur	0.7165	0.7704	0.8486	0.7782	**0.9139**	0.8460	0.8449	0.7191	**0.8904**
	WN	0.9000	**0.9452**	0.9313	0.9217	0.8904	0.9243	0.9069	0.9226	**0.9374**
	FF	0.8695	0.9165	0.9591	0.9391	**0.9747**	0.9591	0.9565	0.9530	**0.9757**

LIVE II-A	JPEG	0.5737	0.7143	**0.8198**	0.7211	0.6894	0.6807	0.6359	0.6304	**0.7449**
	JP2K	0.6076	0.7265	0.7658	0.7539	**0.8727**	0.6356	0.6901	0.7979	**0.8206**
	Gblur	0.7943	0.8057	0.7724	0.8276	**0.9100**	0.8402	0.6911	0.7733	**0.8694**
	WN	0.7725	0.8821	**0.9330**	0.9026	0.8809	**0.9409**	0.9292	0.8009	0.6289
	FF	0.7659	0.8059	0.7886	0.8405	**0.8913**	0.7856	0.7489	0.7872	**0.8850**
